# Stool DNA testing for early detection of colorectal cancer: systematic review using the HTA Core Model^®^ for Rapid Relative Effectiveness Assessment

**DOI:** 10.3205/000320

**Published:** 2023-06-23

**Authors:** Heidi Stürzlinger, Annette Conrads-Frank, Alexander Eisenmann, Sarah Invansits, Beate Jahn, Andrej Janzic, Marjetka Jelenc, Tatja Kostnapfel, Simona Mencej Bedrac, Nikolai Mühlberger, Uwe Siebert, Gaby Sroczynski, European Network for Health Technology Assessment (EUnetHTA)

**Affiliations:** 1 Austrian Public Health Institute (GOEG), Vienna, Austria; 2 Department of Public Health, Health Services Research and Health Technology Assessment, UMIT TIROL – University for Health Sciences, Medical Informatics and Technology, Hall i.T., Austria; 3 Agency for Medicinal Products and Medical Devices of the Republic of Slovenia (JAZMP), Ljubljana, Slovenia; 4 National Institute of Public Health (NIJZ), Ljubljana, Slovenia

**Keywords:** colorectal neoplasms, multitarget stool DNA test, colorectal cancer screening, sensitivity, specificity, test performance

## Abstract

**Background:**

Stool DNA testing for early detection of colorectal cancer (CRC) is a non-invasive technology with the potential to supplement established CRC screening tests. The aim of this health technology assessment was to evaluate effectiveness and safety of currently CE-marked stool DNA tests, compared to other CRC tests in CRC screening strategies in an asymptomatic screening population.

**Methods:**

The assessment was carried out following the guidelines of the European Network for Health Technology Assessment (EUnetHTA). This included a systematic literature search in MED-LINE, Cochrane and EMBASE in 2018. Manufacturers were asked to provide additional data. Five patient interviews helped assessing potential ethical or social aspects and patients’ experiences and preferences. We assessed the risk of bias using QUADAS-2, and the quality of the body of evidence using GRADE.

**Results:**

We identified three test accuracy studies, two of which investigated a multitarget stool DNA test (Cologuard^®^, compared fecal immunochemical test (FIT)) and one a combined DNA stool assay (ColoAlert^®^, compared to guaiac-based fecal occult blood test (gFOBT), Pyruvate Kinase Isoenzyme Type M2 (M2-PK) and combined gFOBT/M2-PK). We found five published surveys on patient satisfaction. No primary study investigating screening effects on CRC incidence or on overall mortality was found. Both stool DNA tests showed in direct comparison higher sensitivity for the detection of CRC and (advanced) adenoma compared to FIT, or gFOBT, respectively, but had lower specificity. However, these comparative results may depend on the exact type of FIT used. The reported test failure rates were higher for stool DNA testing than for FIT. The certainty of evidence was moderate to high for Cologuard^®^ studies, and low to very low for the ColoAlert^®^ study which refers to a former version of the product and yielded no direct evidence on the test accuracy for ad-vanced versus non-advanced adenoma.

**Conclusions:**

ColoAlert^®^ is the only stool DNA test currently sold in Europe and is available at a lower price than Cologuard^®^, but reliable evidence is lacking. A screening study including the current product version of ColoAlert^®^ and suitable comparators would, therefore, help evaluate the effectiveness of this screening option in a European context.

## Zusammenfassung

**Hintergrund:**

Stuhl-DNA-Tests zur Früherkennung des kolorektalen Karzinoms (KRK) sind nicht-invasiv und können etablierte KRK-Screening-Verfahren ergänzen. Ziel dieses Health Technology Assessment war die Untersuchung der Wirksamkeit und Sicherheit von CE-zertifizierten Stuhl-DNA-Tests im Vergleich zu anderen Tests für ein Screening einer asymptomatischen KRK-Screening-Population.

**Methodik:**

Das Assessment wurde nach den Richtlinien des Europäischen Netzwerks für Health Technology Assessment (EUnetHTA) durchgeführt und schloss eine systematische Literaturrecherche in MEDLINE, Cochrane und EMBASE ein, durchgeführt 2018. Die Hersteller wurden bezüglich der Übermittlung von weiteren Daten kontaktiert. Fünf Patienteninterviews halfen in der Einschätzung möglicher ethischer oder sozialer Aspekte sowie von Patientenerfahrungen und -präferenzen. Wir bewerteten das Verzerrungsrisiko mit QUADAS-2 und verwendeten GRADE, um die Qualität der Evidenz zu bewerten.

**Ergebnisse:**

Wir identifizierten drei Studien zur Testgenauigkeit; zwei untersuchten einen Multitarget-Stuhl-DNA-Test (Cologuard^®^, im Vergleich zu einem fäkalen immunchemischen Test (FIT)) und eine Studie einen kombinierten DNA-Stuhltest (ColoAlert^®^, im Vergleich zu einem guajakbasierten Stuhlbluttest (gFOBT), Pyruvate Kinase Isoenzyme Typ M2 (M2-PK) und kombiniertem gFOBT/M2-PK). Wir fanden fünf publizierte Erhebungen zur Patientenzufriedenheit, jedoch keine Primärstudien zu den Auswirkungen eines Screenings mit den beiden Tests auf KRK oder die Gesamtmortalität. Beide Stuhl-DNA-Tests zeigten im direkten Vergleich eine höhere Sensitivität für den Nachweis von KRK und (fortgeschrittenen) Adenomen als FIT beziehungsweise gFOBT, wiesen aber eine geringere Spezifität auf. Diese Ergebnisse könnten jedoch vom genauen Typ des jeweils verwendeten FIT abhängen. Die berichteten Testausfallraten waren beim Stuhl-DNA-Test höher als beim FIT. Die Stärke der Evidenz war moderat bis hoch für die Cologuard^®^-Studien und niedrig bis sehr niedrig für die ColoAlert^®^-Studie, die sich auf eine frühere, nicht mehr am Markt befindliche Version des Produkts bezieht und die in den Ergebnissen zur Testgenauigkeit nicht zwischen fortgeschrittenen und nicht-fortgeschrittenen Adenomen differenzierte.

**Schlussfolgerungen:**

ColoAlert^®^ ist der einzige derzeit in Europa am Markt befindliche Stuhl-DNA-Test und ist zu einem niedrigeren Preis als Cologuard^®^ erhältlich, jedoch fehlt zuverlässige Evidenz. Eine Screening-Studie mit Implementierung der aktuellen Produktversion von ColoAlert^®^ und geeigneten Komparatoren würde daher helfen, diese Screening-Option im europäischen Kontext zu evaluieren.

## Introduction

Colorectal cancer (CRC) is – worldwide and in developed countries – the second most commonly diagnosed cancer in females and the third in males. It is also a leading cause of cancer-related deaths within developed countries [1]. CRC typically develops in pre-existing benign polyps following genetic transformations. In most of the cases, colorectal carcinoma manifest as adenocarcinoma originating from epithelial cells of the colorectal mucosa. In the early stage of disease, many patients have no or non-specific symptoms [2], [3], [4], [5]. Symptoms become more common and prominent during late stages of CRC and include abdominal or back pain, rectal bleeding, iron deficiency anemia, and/or melena, altered bowel habits and shape, weight loss, diarrhea or constipation, nausea and vomiting, malaise, anorexia, and abdominal distention [6], [7], [8].

Due to the natural history of disease with slow progression from a premalignant polyp to cancer and the high incidence and associated mortality, CRC is suitable for population screening [9], [10], [11], [12], [13]. The Council of EU Recommendation recommends CRC screening in a target average-risk population between 50 and 74 years of age. Screening modalities include fecal occult blood testing, either guaiac-based (gFOBT) or immunochemical (FIT). With gFOBT or FIT, most of the established screening programs start between 50 and 60 years of age, with a two-year screening interval. A ten-year interval or more is recommended for screening with endoscopic screening methods, that is flexible sigmoidoscopy or total colonoscopy. It is recommended to continue screening up to the age of 70 to 75 years [14], [15].

With regard to test performance characteristics, FIT is seen as superior to gFOBT. According to guidelines, combining flexible sigmoidoscopy with a stool-based test yields better results than either test alone [16]. (Total) colonoscopy is considered the reference standard for the detection of CRC, allowing an examination of the complete colon (albeit it might overlook small tumours). It is used both as a primary screening tool and as a follow-up for patients who have tested positive [16], [17], [18], [19], [20], [21], [22]. Colonoscopy participation rates, however, often are not seen as sufficient, whereas non-invasive screening tests might yield higher compliance.

Non-invasive deoxyribonucleic acid (DNA) stool tests have been developed for early screening and prevention of CRC. The expected benefit is that they might be superior to the other non-invasive screening tests in terms of test accuracy and comparable in terms of patient compliance. They are usually combined with FIT or gFOBT and are designed for detection of tumour DNA in the stool. Two stool DNA tests in Europe have a CE-mark as of 2018, ColoAlert^®^ (PharmGenomics) and Cologuard^®^ (Exact Sciences). Only ColoAlert^®^ is currently sold in Europe. It is a combination of two tests:

1. a FIT (test in fecal occult blood detecting globin by immunochemical reactions), and
2. a DNA test detecting three molecular genetic markers in stool DNA: mutations in BRAF and KRAS, and quantification of human DNA (hDNA).

In June 2020, the manufacturer website [23] gave a price of 649 USD for Cologuard^®^ (around 578 EUR as of June 2020). Since an update the manufacturer website no longer publishes a price in the FAQs [24] but directly refers to reimbursement of the product [25].

In 2022 from the manufacturer’s online shops in Germany and Austria ‘ColoAlert Basic’ can be ordered at a price of € 139.95 EUR in Austria [26] and ‘ColoAlert Stuhltest’ can be ordered at a price of € 159.95 EUR in Germany [27]. The Austrian price excludes value added tax and shipping costs.

## Research question

The aim of the study was to assess the effectiveness and safety of stool DNA testing for early detection of colorectal cancer compared to other tests and to assess potential ethical, organisational, social and legal issues. Detailed research questions (see Methods section) also included patient satisfaction with the test. Table 1 (Tab. 1) shows the defined PICOS (Population, Intervention, Comparison, Outcomes, Study designs) criteria.

## Methods

### Methodological framework

Methods followed the guidelines of the European Network for Health Technology Assessment (EUnetHTA) for Rapid Relative Effectiveness Assessments and are described in detail in the full assessment report [28], which is available from the website of EUnetHTA. Detailed research questions were formulated according to the HTA Core Model^®^ for Rapid Relative Effectiveness Assessment Version 4.2 [29] (including potential ethical, organisational, social and legal issues), and additional questions according to the HTA Core Model^®^ Version 3.0 [30], Application for Screening Technologies, were added if applicable.

To assess the short- and long-term benefits as well as unintended harms of stool DNA screening strategies in comparison to strategies using alternative tests (e.g. colonoscopy, FIT) a benefit-harm analysis applying a decision-analytic model was conducted in addition. This analysis is described elsewhere [28].

### Literature search and selection

We conducted a systematic literature search in MEDLINE, the Cochrane Library and EMBASE in August 2018. In October 2018, a primary study [31] with an abstract publication from 2016 [32] was published as a full-text article and was added to the study pool as the only study on ColoAlert^®^. We searched for ongoing studies in clinical trial registries (ClinicalTrials.gov, World Health Organization International Clinical Trials Registry Platform and the EU Clinical Trials Register) with an update search in March 2019. We performed a manual search in addition to the systematic search.

Two of the authors screened abstracts independently from each other for inclusion and exclusion, based on the predefined PICOS criteria (Table 1 (Tab. 1)). The same criteria were applied for the full text screening of selected abstracts, performed by the same two authors independently from each other, with cases of dissent being discussed between them. We restricted language to English or German. We checked all relevant systematic reviews and meta-analyses for additional primary studies not identified by the systematic search and screened all abstracts for literature that might be relevant for epidemiologic and technology issues.

### Data extraction and quality assessment

One author extracted all relevant data of the included test accuracy studies. Results were checked by another author. We assessed risk of bias by using Quality Assessment of Diagnostic Accuracy Studies 2 (QUADAS-2 [33]), carried out by two authors independently of each other, with discrepancies resolved by consensus. We additionally assessed the quality of the body of evidence using Grading of Recommendations, Assessment, Development and Evaluation (GRADE).

### Stakeholder involvement

Manufacturers of the two tests were contacted regarding contribution of data. One gave a (positive) reply and submitted device-specific information via the EUnetHTA submission file as well as answers on further queries regarding the manufacturer-sponsored study on ColoAlert^®^.

Patients or healthy individuals were involved during the scoping phase via interviews (telephone or face to face). Five persons, fulfilling the criteria for a CRC screening population experienced with DNA stool testing, gFOBT, FIT or colonoscopy, were identified, either by personal communication or via a physician’s office. A standardised open questionnaire was used asking them about their experiences and preferences regarding screening tests [28]. We used information from patient involvement for assessing the relevance of potential ethical and social aspects and for answering research questions related to patient aspects (e.g. satisfaction with the test).

## Results

### Search results

**Figure 1:**
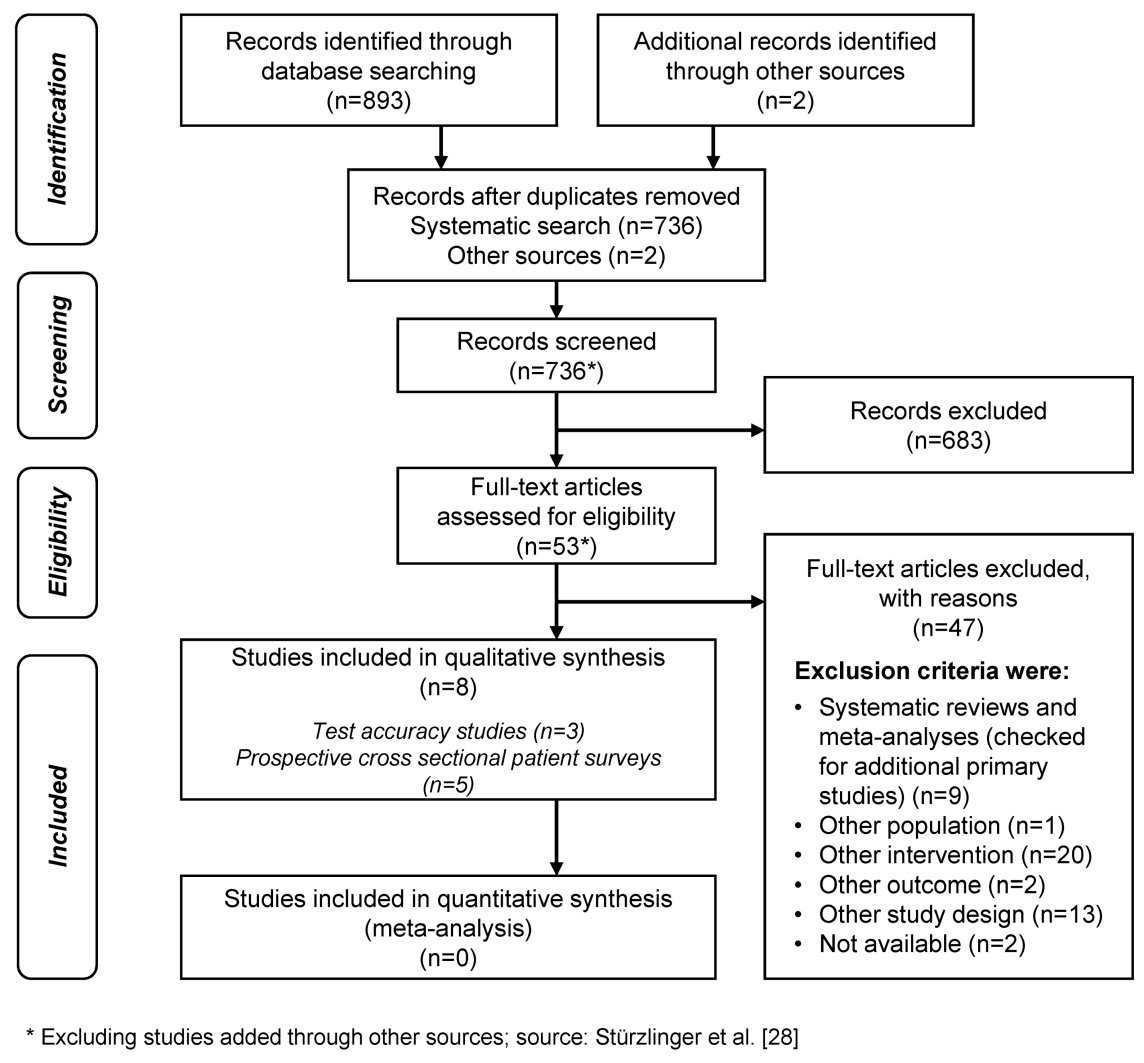
PRISMA flow chart of the study selection process

Figure 1 (Fig. 1) shows the study selection process. Out of the eight included studies, three investigated test accuracy; two of them assessed Cologuard^®^ [34], [35] and one study assessed ColoAlert^®^ [31] (Table 2 (Tab. 2)). Five published patient surveys [36], [37], [38], [39], [40] investigating patient perceptions and preferences of CRC screening tests including stool DNA testing were identified via systematic literature search, but only one of them investigated one of the currently available tests (Cologuard^®^) [36]. They were used to complement the results from the patient interviews. No primary study was identified assessing the effectiveness of DNA stool tests on CRC incidence, CRC mortality, overall mortality or health-related quality of life.

### Study characteristics

Imperiale et al. [35] conducted a cross-sectional screening study across 90 sites throughout the USA and Canada with recruitment lasting from June 2011 through November 2012. They compared the Cologuard^®^ DNA stool test with a FIT [OC FIT-CHEK^®^ (Polymedco)]. In a prospective screening cohort study, Brenner et al. [34] assessed the diagnostic performance of another FIT [FOB Gold^®^ (Sentinel Diagnostics)] and – with adjusted cut-off – compared it with performance data of Cologuard^®^, as reported by Imperiale et al. [35]. Recruitment took place in 20 gastroenterology offices in Southern Germany from November 2008 to September 2014. Dollinger et al. [31] compared in a preclinical case cohort study a combined DNA stool assay [ColoAlert^®^ combined with a gFOBT and an hDNA quantification test (threshold 15 ng/µL)] with a single gFOBT (ColoScreen-ES^®^, Helena Biosciences), a single tumour Pyruvate Kinase Isoenzyme Type M2 (M2-PK) test (ScheBo Biotech AG) and a combined gFOBT/M2-PK assay. They recruited patients from 16 different sites in Germany from August 2005 to May 2007. Detailed study characteristics can be found in Table 2 (Tab. 2).

Five prospective cross-sectional patient surveys from USA [36], [37], [38], [39], [40] were performed in (asymptomatic) screening populations, some of these study populations with and some without previous CRC screening experience. Four of these studies [37], [38], [39], [40] referred to a USA precursor test (PreGen-Plus^®^) of Cologuard^®^, which is no longer available [41]. Only one survey [36] investigated Cologuard^®^, comparing colonoscopy with DNA stool testing (for further details see the full assessment Report [28]).

### Risk of bias for test accuracy studies

For the two studies investigating Cologuard^®^ [34], [35], we noted a risk of bias regarding patient selection (Table 3 (Tab. 3)), no other concerns arose. We noted a considerable risk of bias as well as applicability concerns for the study investigating ColoAlert^®^ [31] (Table 3 (Tab. 3)). Concerns were high that the study population did not match well with the research question of this assessment. Moreover, the stool DNA assay evaluated in the study was different from the currently available product regarding several components.

### Patient interviews

Five individuals (three female/two male) at the age of 56 to 65 were included. All of them were living in Austria. Summarised results are shown in Table 4 (Tab. 4).

### Effectiveness outcomes

Table 5 (Tab. 5) details test accuracy results for the detection of CRC and of adenoma, which are divided into advanced precancerous lesions (APL) and Non-APL. For the detection of CRC, Cologuard^®^ showed a sensitivity of 92.3% (compared with 73.8% and 96.7% for OC FIT-CHEK^®^ and FOB Gold^®^, respectively) and 46.4% for the detection of CRC or APL (compared with 27.7% and 51.1% for OC FIT-CHEK^®^ and FOB Gold^®^, respectively). The specificity for the detection of CRC was 84.4% (compared with 93.4% and 83.0% for OC FIT-CHEK^®^ and FOB Gold^®^, respectively) and 86.6% for the detection of CRC or APL (compared with 94.9% and 86.5% for OC FIT-CHEK^®^ and FOB Gold^®^, respectively). For ColoAlert^®^ the sensitivity to detect CRC was 84.6% (compared with 68.0% and 82.9% for gFOBT and M2-PK, respectively). The sensitivity for this test was 35.5% for the detection of CRC or (any) adenoma (compared with 22.3% and 54.7% for gFOBT and M2-PK, respectively), without discriminating APL from Non-APL. Its specificity was 87.0% for the detection of CRC (compared with 95.5% and 58.7% for gFOBT and M2-PK, respectively) and 88.4% for the detection of CRC or adenoma (compared with 95.8% and 60.1% for gFOBT and M2-PK, respectively). Calculations of positive and negative predictive values as well as of number needed to screen can be found in the full report [28].

### Safety outcomes

No reports of adverse events or user-dependent harms of DNA stool tests were found (or mentioned) within the identified primary evidence. We also found no studies that directly investigated the consequences of false positive or false negative test results from the viewpoint of patient safety [28].

### Other outcomes

Test failures include tests that have not been submitted or that are unevaluable or unusable. The test failure rates were 6.25% for Cologuard^®^ and 0.31% for OC FIT-CHEK^®^ (Table 6 (Tab. 6)). For the study including ColoAlert^®^ only a combined failure rate of all stool tests investigated was available, which amounted to 17.74% (Table 6 (Tab. 6)).

Handling problems carrying out the test and/or taking the specimen were reported by four of the five persons interviewed for this study. Difficulties with having bowel movements were reported once. Results of the five identified published patient surveys do not hint at major handling problems for the majority of patients (for details see Stürzlinger et al. [28]).

Regarding patient preferences, four of the five interviewees said they would rather do the experienced stool test (FIT in two persons and gFOBT in the two other) than colonoscopy (three of them had already undergone a colonoscopy). One person, who was experienced in all of the four tests, appeared to be indifferent. Rather inconsistent results on screening test preferences were found within the five identified published patient surveys (for details see Stürzlinger et al. [28]).

### Organisational aspects

Most stool tests can be ordered via the Internet or bought in a pharmacy. Cologuard^®^ is available by prescription only [42], [43]. Users can administer stool tests at home, but specimens (mostly) have to be sent to a specialiced laboratory for analysis.

No (further) relevant ethical, social or legal aspects were identified.

## Discussion

Of the two CE-marked DNA stool tests, ColoAlert^®^ is the most recent product, being authorised in 2016. It is the only DNA stool test currently sold on the European market. In our systematic literature search we identified three test accuracy studies, two on Cologuard^®^ (both referring to the same Cologuard^®^ study population [34], [35]) and one [31] investigating ColoAlert^®^. The certainty of evidence was moderate to high for Cologuard^®^ results and low to very low for ColoAlert^®^ results [28]. Besides serious concerns about patient selection (Table 3 (Tab. 3)), recruitment of the study dates back to 2005 to 2007 and a former version of the test was used that differs in several components from the currently available product. Also the study did not report information on the exact proportion of test failures in the DNA assay alone compared with the other stool tests [28].

The test accuracy (against the reference standard) of CRC triage screening tests cannot easily be depicted as one value for sensitivity and one for specificity. Not all precancerous lesions – if not removed – progress to clinically symptomatic cancer [44], [45]. Thus triage screening tests should yield a positive test result in persons with CRC and, preferably, also in persons with advanced adenomas (which can be removed by polypectomy and should be followed by shorter surveillance intervals thereafter). On the one hand, it might be debated if they should also yield a positive result (and, thus, reference to colonoscopy) in cases of non-advanced adenomas. On the other hand, with regard to specificity, either the proportion of negative test results in all persons without CRC or (any) adenoma, or the proportion of negative test results in all persons without CRC or advanced adenoma, is of interest. This differentiation, however, was not reported in the study by Dollinger et al. [31], making it difficult to interpret and compare the test accuracy results. For the detection of CRC, ColoAlert^®^ yielded a lower sensitivity than Cologuard^®^, and, on the other hand, correctly detected a higher proportion of completely healthy persons (Table 5 (Tab. 5)). Remarkably, the test accuracy results of FIT differed largely, depending on brand and cut-off value. Though this was not a focus of this assessment, it might be a relevant issue for comparison. There was no direct comparison between ColoAlert^®^ and FIT. Lastly, also test failure rates are a relevant issue for judging test accuracy. Test failures can partly be compensated by collecting a second specimen, although this is associated with increased time effort and potential costs. Only in one study [35], test failure rates were completely reported, and were highest for stool DNA testing, followed by colonoscopy, and FIT.

Results of this HTA are limited by the fact that not all PICO-comparators were investigated within the identified studies, which also is connected to the very small number of studies available for the CE-marked products. Also, the incorporation of patient views was limited by the difficulty of finding patients that had stool DNA test experience. Patient surveys found in the literature mostly referred to a precursor test of Cologuard^®^.

In our systematic literature search, we did not identify studies on long-term effects of stool DNA tests on mortality and morbidity, which might be due to the short time period DNA tests are on the market. With regard to adverse events or direct user-dependent harms, no major findings were reported. Undoubtedly, there will be consequences from false positive and false negative test results as undetected adenomas, on the one hand might progress further and false positive results, on the other hand, lead to unnecessary colonoscopies. Moreover, positive test results mostly lead to immediate worry and all of the test procedures, but namely colonoscopies, imply some kind of immediate burden to the person tested. The benefit-harm tradeoff of respective screening strategies was investigated within a decision-analytic modeling done for this assessment [28], but not reported in this article.

Finally, the literature search for this HTA was done in 2018. An update systematic rapid review published in 2021 [46] which was based on the original literature search of our report [28] found two further studies on Cologuard^®^ and concluded that these newer studies confirm the existing results regarding diagnostic test accuracy, with additional (favourable) results on the specificity of Cologuard^®^ for detecting CRC in persons 45 to 49 years old [46].

## Conclusions

Overall, stool DNA tests showed higher sensitivity for the detection of CRC and (advanced) adenoma than FIT or gFOBT, but lower specificity. The results depended to a degree on the exact type of FIT used. The reported test failure rate of stool DNA tests was higher than that of FIT.

ColoAlert^®^ is the only stool DNA test currently sold in Europe and is available at a lower price than Cologuard^®^. Reliable evidence on ColoAlert^®^ is lacking, however. A cross-sectional screening study including the current product version, as well as FIT as additional comparator, would therefore help in evaluating this screening option in a European context. In terms of the comparator tests, especially FIT, it would be desirable to carefully select the brand and especially the cut-off value and provide some rationale for those choices. Also, (directly) addressing the effectiveness of DNA stool tests on morbidity, mortality and health-related quality of life, by conducting prospective (randomised) controlled trials, should be considered.

## Abbreviations

- APL: Advanced precancerous lesions
- CRC: Colorectal cancer
- DNA: Invasive deoxyribonucleic acid
- EUnetHTA: European Network for Health Technology Assessment
- FIT: Fecal immunochemical test
- gFOBT: Guaiac-based fecal occult blood test
- GRADE: Grading of Recommendations, Assessment, Development and Evaluation
- HTA: Health technology assessment
- M2-PK: Pyruvate Kinase Isoenzyme Type M2
- PICOS: Population, Intervention, Comparison, Outcomes and Study designs
- QUADAS-2: Quality Assessment of Diagnostic Accuracy Studies 2

## Notes

### Acknowledgements

The authors thank Eunate Arana-Arri, Fidencio Bao Pérez, Gerfried Lexer and Isabel Idigoras Rubio for serving as external medical experts in the EUnetHTA Rapid Relative Effectiveness Assessment of stool DNA testing. Furthermore, the authors are grateful to individual experts from the National Institute for Health and Care Excellence (NICE), National Agency for Regional Health Services (AGENAS), Social & Health Services and Labour Market (DEFACTUM) and Basque Office for HTA (Osteba) for the review of the draft EUnetHTA report. EUnetHTA Joint Action 3 was supported by a grant from the European Commission in the framework of the Health Programme (2014-2020; joint action “724130”). The content of this paper represents the views of the authors only and is their sole responsibility; it cannot be considered to reflect the views of the European Commission and/or the Consumers, Health, Agriculture and Food Executive Agency or any other body of the European Union. The European Commission and the Agency do not accept any responsibility for use that may be made of the information it contains.

### Competing interests

See full report [28] (published on the EunetHTA website), page 3.

## Figures and Tables

**Table 1:**
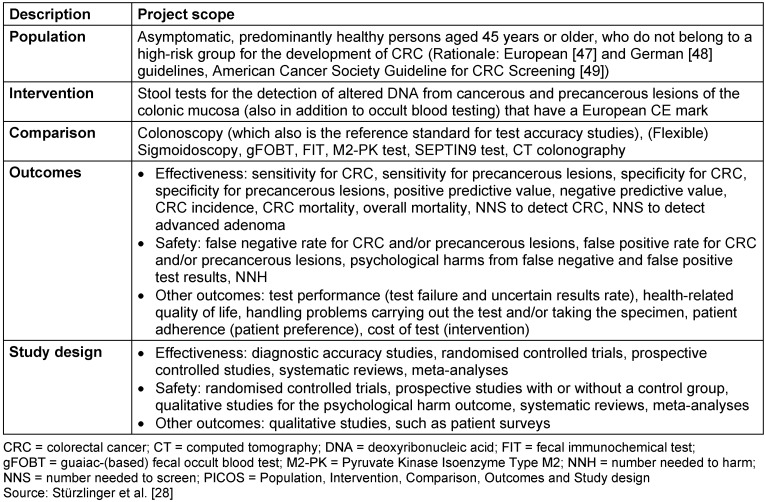
PICOS

**Table 2:**
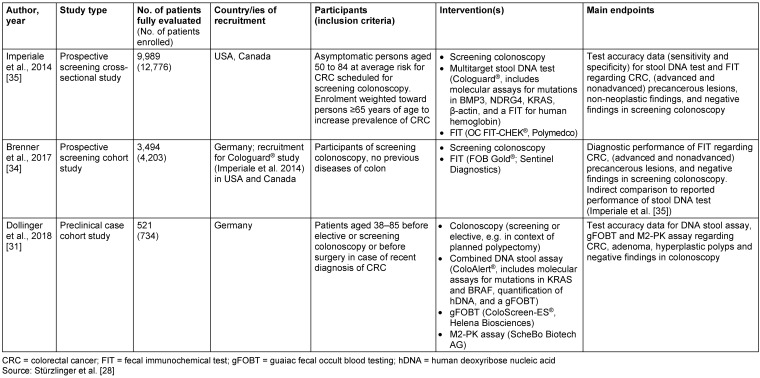
Main characteristics of test accuracy studies included for efficacy and safety

**Table 3:**
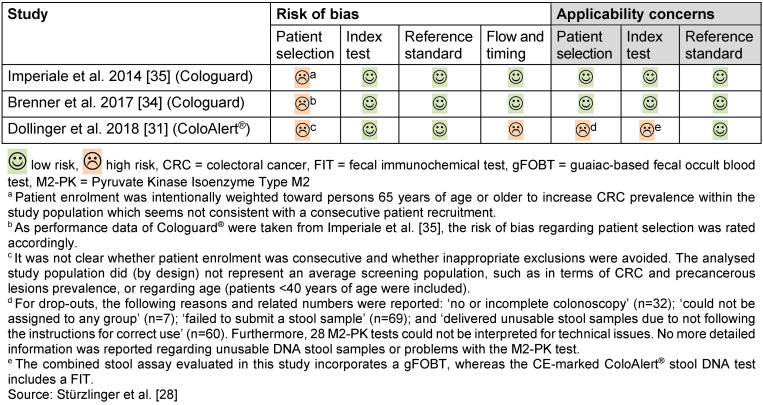
Risk of bias for test accuracy studies (QUADAS-2)

**Table 4:**
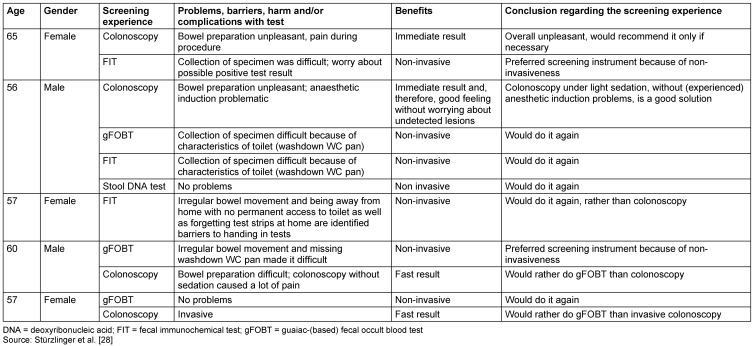
Main results from the five patient interviews

**Table 5:**
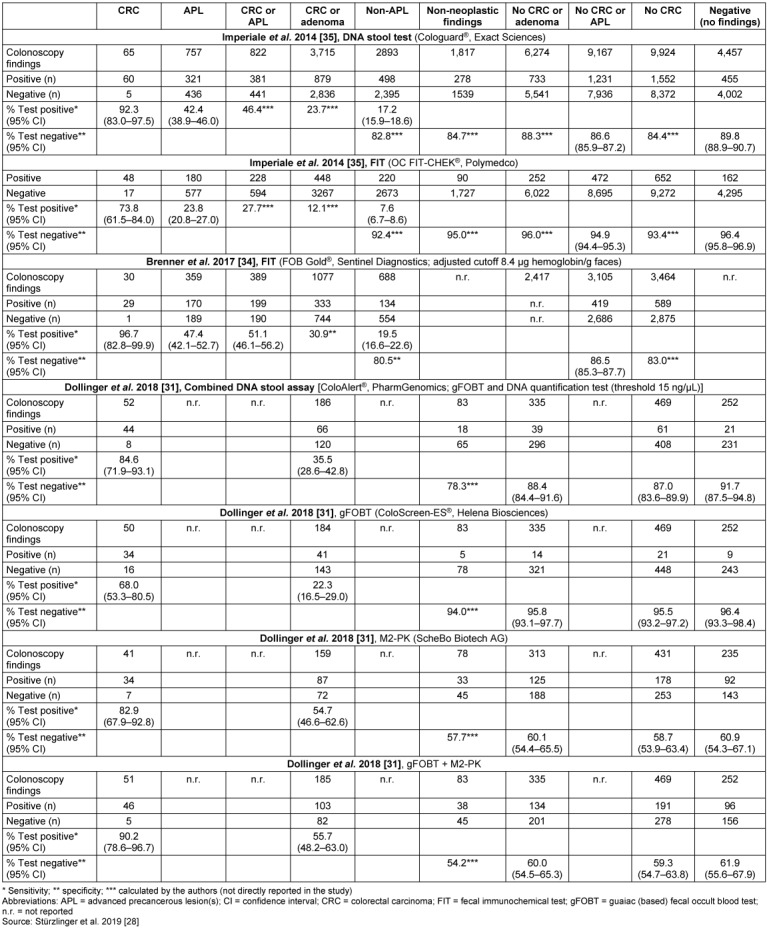
Test accuracy data – sensitivity and specificity

**Table 6:**
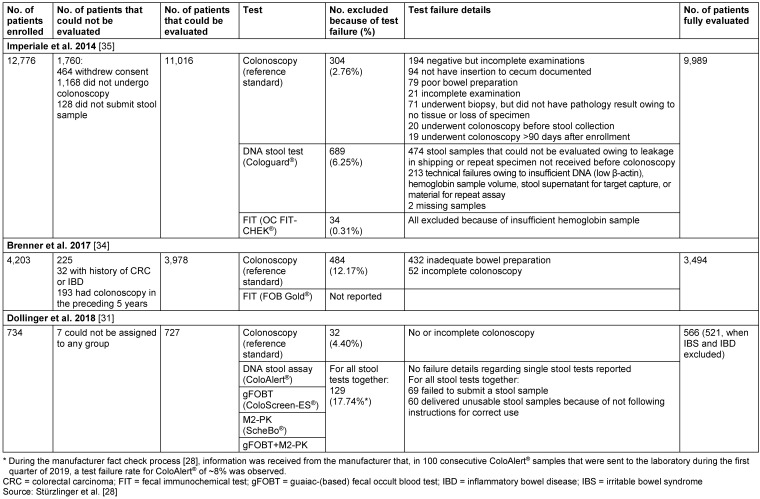
Test performance – failure rates
